# The Role of Ubiquitin-Proteasome System in the Pathogenesis of Severe Acute Respiratory Syndrome Coronavirus-2 Disease

**DOI:** 10.1155/2023/6698069

**Published:** 2023-03-04

**Authors:** Fikadu Seyoum Tola

**Affiliations:** Department of Medical Biochemistry, College of Medicine and Health Sciences, Ambo University, PO. Box. 19, Addis Ababa, Ethiopia

## Abstract

Different protein degradation pathways exist in cells. However, the bulk of cellular proteins are degraded by the ubiquitin-proteasome system (UPS), which is one of these pathways. The upkeep of cellular protein homeostasis is facilitated by the ubiquitin-proteasome system, which has a variety of important functions. With the emergence of eukaryotic organisms, the relationship between ubiquitylation and proteolysis by the proteasome became apparent. Severe acute respiratory syndrome coronavirus-2 (SARS-Coronavirus-2) hijacks the ubiquitin-proteasome system and causes their viral proteins to become ubiquitinated, facilitating assembly and budding. Ubiquitination of the enzyme keratin-38 (E-K38) residue gave the virion the ability to engage with at least one putative cellular receptor, T-cell immunoglobin-mucin (TIM-1), boosting virus entry, reproduction, and pathogenesis. A fraction of infectious viral particles produced during replication have been ubiquitinated. The ubiquitin system promotes viral replication. In order to replicate their viral genome after entering the host cell, viruses combine the resources of the host cell with recently generated viral proteins. Additionally, viruses have the ability to encode deubiquitinating (DUB)-active proteins that can boost viral replication through both direct and indirect means. The SARS-Coronavirus-2 papain-like protease (PLpro) protein is a DUB enzyme that is necessary for breaking down viral polyproteins to create a working replicase complex and promote viral propagation. The ubiquitin-like molecule interferon-stimulated gene 15 (ISG15), which is likewise a regulator of the innate immune response and has antiviral characteristics, can also be broken down by this enzyme. However, limiting the E1-activating enzyme's ability to suppress the ubiquitination pathway prevented virus infection but did not prevent viral RNA genome translation. Numerous investigations have revealed that the use of proteasome inhibitors has a detrimental effect on the replication of SARS-Coronavirus-2 and other viruses in the host cell. Studies have shown that the use of proteasome inhibitors is also known to deplete free cellular ubiquitin, which may have an impact on viral replication and other vital cellular functions.

## 1. Background

In Wuhan, People's Republic of China, pneumonia of unknown origin was first noticed and reported to the World Health Organization (WHO) in December. Severe acute respiratory syndrome (SARS-Coronavirus-2) was the designation given to the illness after it was later determined to be caused by a new coronavirus [1, 2]. On February 11^th^, 2020, the WHO classified coronavirus infection as COVID-19, commonly known as coronavirus disease 2019. Two months after COVID-19 had spread substantially outside of China, with epidemic foci in South Korea and Japan and a sizable outbreak in Italy, the WHO declared it to be a pandemic [3].

Later, the illness spread quickly over the globe and struck practically every nation. Because of this, most global healthcare systems are ill-prepared to handle the increase in patients needing hospitalization without taking further steps to prevent system collapse. There was worry that the fatality rate may have risen because there were not enough intensive care unit beds to treat the most serious cases [4].

The genetic material of SARS-Coronavirus-2 is positive RNA and expresses open reading frames that encode both structural and functional proteins. The membrane, envelope, spike, and nucleocapsid proteins are all structural proteins. The spike protein's S1 subunit aids in ACE2-mediated virus attachment, and its S2 subunit aids in membrane fusion. The leucine, phenylalanine, glutamine, asparagine, and serine amino acids all contributed to SARS-improved Coronavirus-2's ACE2 binding. The viral serine-rich linker region between the N terminal (NTD) and C terminal makes up the N protein (CTD). These terminals are necessary for both viral entrance and post-entry processing [5].

After the host cell is attached, the host transmembrane serine protease 2 primes the S protein (TMPRSS2). The SARS-Coronavirus-2 is internalized through endocytosis, and the endosome releases the viral genome. The viral RNA is translated into two polyproteins (PPs), pp1a and pp1ab, in the cytosol. These polyproteins are then processed further to form sixteen nonstructural proteins (nsp1 to nsp16), which are the constituent parts of the viral replicase-transcriptase complex [6]. The entire viral genome is subsequently reproduced in vesicles that also contain replicate transcriptase complexes (RTC). The SARS-Coronavirus-2 structural and accessory proteins are produced concurrently, and they come together to form the nucleocapsid and viral envelope at the ER-Golgi intermediate compartment, enabling the release of mature virions later [7].

## 2. Ubiquitin Proteasome System

An adult weighing 60 kg produces and degrades about 240 g of proteins each day, the majority of which are intracellular proteins. A failure to accurately regulate this normal protein homeostasis ultimately leads to various pathological conditions [8]. Thousands of proteins are in dynamic balance to maintain protein homeostasis, or proteostasis, which is essential for the majority of cellular and organismal normal function. The biological process of protein, which includes translation, folding, and degradation, determines the entire repertoire of cellular proteins (proteome) of the cells [9].

The ubiquitin-proteasome system of the cell (UPS), which is involved in the breakdown of the majority of cellular proteins, is one of the various protein degradation systems used by cells. In the upkeep of cellular protein homeostasis, the ubiquitin-proteasome system plays a vital and varied role [10]. The pathway to proteasomal degradation involves a group of enzymes called ubiquitin ligases that attach ubiquitin moieties to protein substrates [11].

More than 80% of cellular proteins, particularly short-lived, regulatory, damaged, and misfolded proteins, are degraded by the UPS, which accounts for the regulation of many cellular functions. The ubiquitin-proteasome system controls numerous biological processes, such as the cell cycle, gene transcription, translation, cell survival, cell death, cell metabolism, protein quality, and inflammation, by degrading particular proteins that are involved in these processes [12].

Due to the extreme specificity of UPS's substrate recognition, individual proteins must first be tagged before being sorted. The proteasome produces a highly heterogeneous combination of short and long peptides rather than breaking down proteins into their constituent amino acids. These peptides are either used as a substrate for the immune system that is mediated by adaptive cells or further processed into amino acids by cytosolic peptidases for the de novo synthesis of proteins [13].

## 3. Ubiquitylations

Since the biological method of protein degradation (proteolysis) is irreversible, numerous complex mechanisms have been developed to ensure effective and selective protein degradation [14]. With the emergence of eukaryotic organisms, the relationship between ubiquitylation and proteolysis by the proteasome became apparent. The ubiquitin-proteasome system is made up of the ubiquitin system, which breaks down ubiquitylated proteins, and the ubiquitylation system, which uses specific enzymes to ubiquitylate or deubiquitylate target proteins [15]. Three enzymes, including E1, E2, and E3, are required for the sequential, adenosine triphosphate (ATP)-dependent process of ubiquitylation. These processes involve the covalent attachment of monomers (ubiquitylation) or polyubiquitylations of highly conserved ubiquitin proteins to protein substrates [16].

In all mammalian cells, ubiquitin is activated by the first enzyme (E1), known as the ubiquitin-activating enzyme. A new covalent link is formed between the carboxyl-terminal of ubiquitin proteins and the lysine, cysteine, serine, or methionine residues of the target proteins by the E2 ubiquitin conjugation enzyme after activation [17]. An enzyme E3, of the ubiquitin ligase family, typically contributes to the conjugation process as well, acting as a docking platform to join the substrate and the ubiquitin-charged E2, which occasionally involves ubiquitin conjugation to a cysteine in the E3 enzyme during transfer to the substrate [17].

Contrary to proteolysis, ubiquitylation of proteins is a reversible process, and the deubiquitylating enzyme closely controls the removal of ubiquitin from the substrate. Before proteasomal degradation, DUBs release polyubiquitin chains from substrates and cleave ubiquitin from proteins [18]. Deubiquitylating enzyme activity is crucial for recycling ubiquitin for subsequent reactions, avoiding congestion, and managing protein turnover by altering or eliminating ubiquitin or polyubiquitin chains from the targeted protein, preventing proteasomal degradation [19].

## 4. The Ubiquitin-Proteasome and Its Mechanism of Action

The designation of proteins for proteasome-mediated protein degradation is carried out by the 76-amino acid protein ubiquitin. The intricate process of ubiquitination requires a number of enzymes, including the E1 ubiquitin-activating enzyme, the E2 ubiquitin-conjugating enzyme, and the E3 ubiquitin ligase enzyme.

Ubiquitin is activated at this stage through an ATP-dependent process involving the E1 ubiquitin-activating enzyme. After forming a compound with E2 and E1, ubiquitin will be charged to the E2 ubiquitin conjugating enzyme [20].

**Figure 1:**
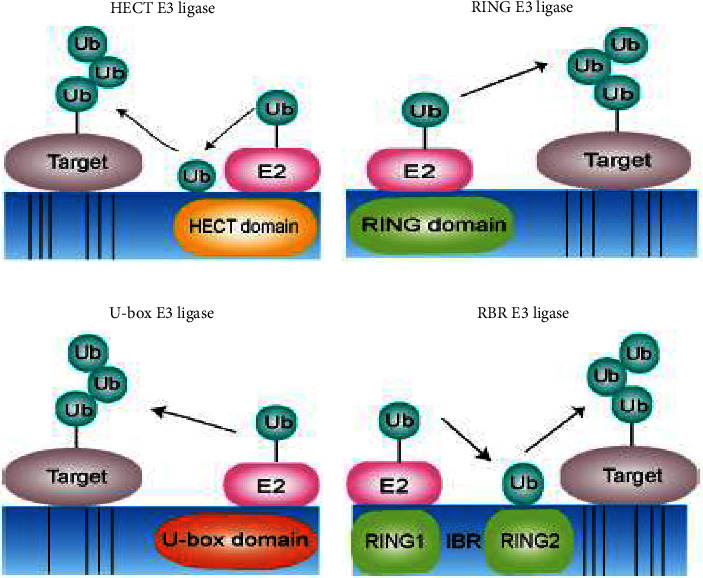
Types of ubiquitination ligases and ligase E3 function. (a) The HECT type of E3 ligases contains the conserved C-terminal HECT domain, and the N-terminal consists of different domains depending on the specific subtype. HECT-type E3 ligases are involved in the ubiquitination process, which involves a two-step reaction: ubiquitin is first carried out by an E2 ligase binding to the HECT domain of the E3 ligase and then transferred to a catalytic cysteine on the E3 ligase; the second step is the transfer of ubiquitin from the E3 ligase to the substrate. (b) The RING type E3 ligases are characterized by the presence of a zinc-binding domain called a RING at the N-terminal. Ring E3s mediate a direct transfer of ubiquitin from E2 ligase to the substrate. (c) The U-box type E3 ligases contain a U-box domain at the C-terminal, which is responsible for binding the ubiquitin-charged E2 ligase and stimulating ubiquitin transfer. (d) The RBR type E3 ligases consist of two predicted RING domains (RING1 and RING2) separated by an IBR domain. RBR type E3 ligases' catalyzed ubiquitination process involves a two-step reaction where ubiquitin is first transferred to a catalytic RING2 domain on the E3 and then to the substrate [24]. (a) HECT E3 ligase; (b) RING E3 ligase; (c) U-box E3 ligase; and (d) RBR E3 ligase.

Ubiquitin was then transported to a cysteine residue on E2's active site. The homologous to the E6-AP carboxyl terminus (HECT), the retinol-binding protein (RBR), or the really interesting new gene (RING) domain ligases, one of the three kinds of E3 ubiquitin ligases, then interact with this complex to bring in a particular substrate [21]. The HECT and RBR E3 ligases can transfer Ub directly to the substrate by creating an intermediary ubiquitin-thioester on one of the E3 cysteine residues, whereas RING-containing E3 ligases place the Ub-loaded E2 close to the substrate for E2-to-substrate transfer, which typically takes place on a lysine on that substrate [22]. The involvement of the tripartite motif (TRIM) subfamily of E3 ligases in regulating the innate immune response, having both direct and indirect antiviral activity, and promoting viral replication is of particular interest [23] (Figure 1).

The cytoplasm and nucleus of mammalian cells contain the 26 svedberg (S) proteasome, a large, multisubunit proteolytic apparatus. Two 19S regulatory caps and a cylindrical 20S catalytic core made up the 26S proteasome [25]. The barrel-shaped core portion (20S) is created by stacking axially four heptameric rings: two identical inner ß-rings, each made up of seven distinct ß-subunits, and two identical outside -rings, each made up of seven distinct a-subunits, 1–7. A 19S regulatory particle (RP), which caps the 20S CP at each end, controls the protease core's activity [26].

**Figure 2:**
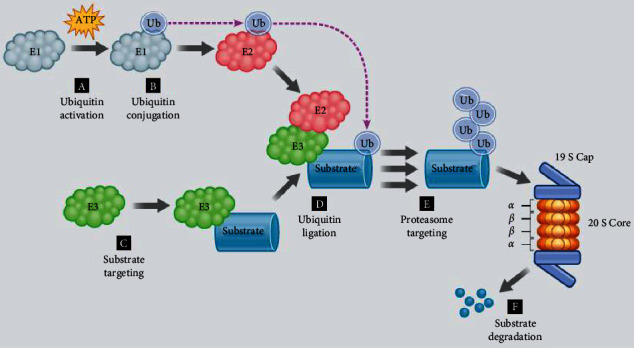
(a) In the presence of ATP, ubiquitin is activated and attached to E1; (b) ubiquitin is conjugated to E2; (c) E3 targets proteins that are intended for destruction; (d) ubiquitin is ligated to the protein substrate; (e) repeated ubiquitin ligation aims the substrate at the proteasome; (f) the 19S cap of the proteasome recognizes the substrate, which is then broken down after entering the 20S core [28].

A number of alpha subunits prevent target proteins from entering the core. These alpha subunits' conformational changes are caused by the 19S regulatory, which also permits the degradation substrate to pass through. The core is made up of three pairs of beta subunits, which also contain the active sites for degradation [27]. The proteolytic activities are trypsin-like (ß1), which cleaves after basic residues, chymotrypsin-like (ß5), which cleaves after hydrophobic residues, or caspase-like (ß2), which cleaves after acidic residues. Threonine residues, which are essential for degradation, are present in each active site. After disintegration, 3–22 amino acid protein fragments with an average length of 7–8 residues flow out the opposite opening of the cylindrical catalytic core and are recycled by the cell for de novo protein synthesis [28] (Figure 2).

## 5. Ubiquitin System and Virus Entry to the Host Cell

Although some viruses can also use less-specific entrance pathways by interacting with lipids or carbohydrates on the surface of the cell membrane, viruses often enter the host cell through highly specific interactions with cellular surface receptors [29]. Enveloped viruses fuse with the cell after attachment, either at the cell surface or the endosomal membrane. Every viral family has a unique entrance mechanism, and SARS-Coronavirus-2 viruses have exploited ubiquitin to speed up this mechanism [30].

The ubiquitin system increases viral propagation. In order to replicate their viral genome after entering the host cell, viruses combine the resources of the host cell with newly produced viral proteins. In order to facilitate entry into the host cell, viruses manipulate the normal cellular ubiquitination process in a proteasome-dependent manner. According to a previous study, the infectious virion of some flaviviruses may contain viral proteins that are ubiquitinated to facilitate extracellular interactions with receptors [31]. The amount of ubiquitinated E found in infectious viral particles produced during replication allowed the virion to connect with at least one possible cellular receptor, TIM-1, facilitating viral entrance, replication, and pathogenicity [32].

Numerous studies have shown that using proteasome inhibitors has a detrimental impact on the virus ability to replicate in the host cell for the human immunodeficiency virus, SARS, and other viruses [33]. Studies indicated that using proteasome inhibitors is also known to deplete free cellular ubiquitin, which may also have an impact on viral replications and other crucial cellular functions [34]. As a result, it was discovered that proteasome inhibitors such as MG132 could prevent the release of viral components into the cytoplasm of the host cell by encouraging the accumulation of viral RNA in the endosome [34, 35].

The proteasome inhibitors reduce internalization by preventing the release of viral ribonucleoproteins (RNP) from endosomes, but they have no effect on the viral attachment or RNA translation [36]. However, because ubiquitination is a crucial mechanism for viral uncoating, inhibiting the E1-activating enzyme prevented the translation of the viral RNA genome rather than preventing virus internalization [37].

In this instance, the ubiquitination of E1 serves as a tissue tropism mechanism in addition to playing a role in the early stages of virus host entrance. Strategies to restrict virus entry might include ubiquitination and the proteasome-dependent destruction of cellular proteins. A medication known as halofuginone was discovered in a screen to activate the E3 ubiquitin ligase complex DNA damage-binding protein (DDB1)-cullin family of ubiquitin ligase (CUL4)-associated factor DCAF1 to promote TMPRSS2 proteasomal degradation. A serine protease called TMPRSS2 facilitates the entry of SARS and SARS-Coronavirus-2 by cleaving the coronavirus spike protein, which is necessary for the virus to bind to the cell. Other stages of the SARS-Coronavirus-2 replication cycle have also been proposed to be inhibited by proteasome inhibitors [38, 39]. Several studies have suggested using proteasome inhibitors to stop the cycle of viral protein replication. Viruses can assemble and budding by ubiquitinating their viral protein using the ubiquitin system as a tool [40].

## 6. The Ubiquitin System and Viral Evasion of the Type-I Interferon (IFN-I) Response

The innate immune system can detect viral invasion to prevent viral replication and infection, making it the initial line of defense against pathogens. When pathogen-associated molecular pattern (PAMP) receptors, which include Toll-like receptors (TLRs) and cytoplasmic RIG-I-like receptors, identify microbial components encoded in bacteria, innate immunity is triggered (PAMPs) [41]. When viral products are recognized by PRRs, this sets off a variety of downstream signaling cascades by activating kinases such as IKK, TBK1, TAK1, and others. IRF3, IRF7, and NF-B are only a few of the transcription factors that are phosphorylated, which facilitate their movement into the nucleus and cause the generation of proinflammatory cytokines such as IFN-I [42, 43].

In order to produce IFN-I, RIG-I must be ubiquitinated by the E3-ubiquitin ligase TRIM25. This activation then triggers downstream signaling via the adaptor protein MAVS, which is likewise heavily regulated by the ubiquitination process [44]. IFNs are released from infected cells, detected by their receptors in an autocrine or paracrine manner, which activates the JAK-STAT signaling cascade and causes the expression of antiviral host effector proteins known as IFN-stimulated genes [45].

Coronaviruses have the capacity to subvert the immune response of the host, including by inhibiting IFN synthesis and signaling, which can boost viral proliferation. The nucleocapsid (N) and papain-like protease (PLpro) proteins from both the original epidemic strain of SARS-Coronavirus-2 and the novel SARS-Coronavirus-2 can inhibit the IFN response. These proteins are encoded by the open reading frames 6 and 7a (ORF6 and ORF7a), respectively [46, 47].

On the other hand, STAT2 phosphorylation is suppressed by the ORF7a protein of SARS-Coronavirus-2, which inhibits IFN-I signaling via its K63-linked polyubiquitination on K119. The SARS-Coronavirus-2 can target TRIM25, another component of the ubiquitin system involved in IFN generation. It is well known that the E3 ligase TRIM25 ubiquitinates RIG-I to cause IFN to be produced later [48, 49].

Viruses have the ability to encode DUB-active proteins, which can improve viral replication through both direct and indirect processes. The processing of viral polyproteins to create a functional replicase complex and promote viral dissemination calls for the SARS-Coronavirus-2 PLpro protein, a DUB enzyme. ISG15, a ubiquitin-like protein that regulates the innate immune response and has antiviral effects, and ubiquitin are both capable of being broken down by this enzyme [50, 51].

Pharmacological suppression of the SARS-Coronavirus-2 ubiquitination proteins can lessen cytotoxic effects while preserving IFN-I responses and limiting virus multiplication. It may also be possible to inhibit nsp3 self-processing to prevent virus reproduction. The NLRP3 inflammasome is activated when the previous pandemic strain of SARS-Coronavirus-1 infects a person, and this could help the virus spread more effectively in vivo. Ubiquitin is involved in this process [52, 53].

When ubiquitin is added to protein substrates, it acts as a highly specific destruction tag, designating the proteins for proteasome disintegration. A method for removing ubiquitin from marked proteins using a different set of enzymes. While poly-Ub chains with Ub moieties joined via iso-peptide bonds at lysine residue 48 (K48) of Ub are frequently used for proteasome targeting, additional Ub linkages, particularly K63, and Ub-like modifications are also frequently used [54].

Many viruses, including SARS-Coronavirus-2, frequently modulate ubiquitin and ubiquitin-like modifiers to avoid the host cell-immune response. Each member of the Coronaviridae family has the genetic code for the viral papain-like protease (PLPs), a deubiquitylating enzyme that removes ubiquitin from target proteins and modifies cellular pathways crucial for viral infection [55]. This enzyme is multifunctional and contains intrinsic cysteine protease activity that is necessary for viral replication and evading host cell defenses in addition to deubiquitylation activity [56].

Similar to DUB, papain-like protease delSGylating involves stripping tagged proteins of their interferon (IFN)-stimulated gene (ISG)-15 moieties. ISG15 is a little peptide that resembles Ub and can be covalently bonded to target proteins in a manner similar to Ub. This can have a variety of regulatory consequences. ISG15 serves as an effector and regulator of the host cell's innate immune response during viral infection and is most increased during antiviral responses [57, 58]. During the early replication stage of coronavirus infection, PLPs play their first role. Once the virus enters the host cell, a replication/transcription complex (RTC) is required to coordinate the replication of the viral unit in the host cytoplasm [59].

SARS and other coronavirus infections frequently include immune system dysregulation as a pathogenic characteristic at the systemic level. PLPs are thought to contribute to infection pathogenesis by leveraging their intrinsic DUBs and delSGylating activities to counteract the activation of the host cell's innate immune response [60, 61].

PLPs specifically exploit their DUB activity to disrupt the immunological pathways, such as the IRF3 and NF-kB pathways, which in turn reduces the antiviral response by interfering with the proteins that underlie intracellular sensing and signaling of viral infection. PLPs typically exhibit broad-spectrum DUB activity rather than DUB activity specific to ubiquitin linkage types K48 or K63 [62].

In the early stages, following SARS-Coronavirus-2 infection, hypoxemia, a decrease in T cells, and an inflammatory storm situation are well-known characteristics of severe COVID-19 patients. The research made the case that hypoxia might increase the mRNA expression of proteasome subunits, which may be related to lymphocyte cell death. Proteasome inhibitors have been shown in numerous studies to induce apoptosis [63].

Additionally, it has been shown that proteasome inhibition causes human monocyte-derived dendritic cells to undergo apoptosis, pointing to a protective role for the proteasome in the immune system [64]. Furthermore, research suggests that viruses and their infected hosts use the proteasomal degradation apparatus to aid in viral replication and prevent viral invasion in the infected host [65]. In this regard, it has been shown that proteasome inhibitors are effective at reducing the viral life cycle, including in SARS-Coronavirus-2 [65, 66].

Due to infection with COVID-19, ubiquitylation balances immune response activation and inhibition [67]. In an in vitro investigation, the expression of 16 genes from the catalytic core and regulatory particles of the 20S proteasome was examined. These results revealed elevated levels of seven proteasome subunits in COVID-19 patients (PSMA4, 5; PSMB2, 9; PSMD14; and PSME1), indicating a potential rise in proteasome activity brought on by subunit overexpression. Future COVID-19 therapy plans may be interested in targeting viral PLPs since coronaviruses need them to both reproduce and counteract the innate immune response. As such, they are important therapeutic targets for coronavirus infections [67, 68].

## 7. Conclusion

A 76-amino acid protein called ubiquitin is well known for its role in designating proteins for proteasome-mediated protein breakdown. A number of enzymes, including the E1 ubiquitin-activating enzyme, E2 ubiquitin-conjugating enzyme, and E3 ubiquitin ligase enzyme, are required for the complex process of ubiquitination. In order to facilitate entry into the host cell, viruses manipulate the normal cellular ubiquitination process in a proteasome-dependent manner. The infectious virion of some viruses may contain viral proteins that are ubiquitinated to facilitate extracellular interactions with receptors on the mechanisms by which the ubiquitination process increases virus entrance.

Coronaviruses have the capacity to subvert the immune response of the host, including inhibiting IFN synthesis and signaling, which can boost viral proliferation. The nucleocapsid (N) and papain-like protease (PLpro) proteins from both the original epidemic strain of SARS-Coronavirus-2 and the novel SARS-Coronavirus-2 can inhibit the IFN response. These proteins are encoded by the open reading frames 6 and 7a (ORF6, ORF7a), respectively.

The amount of ubiquitinated E found in infectious viral particles produced during replication allowed the virion to connect with at least one possible cellular receptor, TIM-1, facilitating viral entrance, replication, and pathogenicity. Numerous studies have shown that using proteasome inhibitors has a detrimental impact on the virus' ability to replicate in the host cell for the human immunodeficiency virus, SARS, and other viruses. Studies indicate that using proteasome inhibitors is also known to deplete free cellular ubiquitin, which may also have an impact on viral replication and other crucial cellular functions.

As a result, it was discovered that proteasome inhibitors like MG132 could prevent the release of viral components into the cytoplasm of the host cell by encouraging the accumulation of viral RNA in the endosome. The proteasome inhibitors reduce internalization by preventing the release of viral ribonucleoproteins (RNP) from endosomes, but they have no effect on viral attachment or RNA translation. However, because ubiquitination is a crucial mechanism for viral uncoating, inhibiting the E1-activating enzyme prevented the translation of the viral RNA genome rather than preventing virus internalization. However, the proteasome inhibitors' effect on SARS-Coronavirus-2 and host cell receptors attachment requires further investigation. Additionally, the effect of proteasome inhibitors on the normal cellular UPS mechanism is not well understood.

## Data Availability

The data supporting this review article are from previously reported studies and datasets, which have been cited.

## Abbreviations

ACE2: Angiotensin converting enzyme-2
CTD: C terminal domain
IFN: Interferon
TMPRSS2: Host transmembrane serine protease 2 primes the S protein
JAK-STAT: Janus kinase-signal transducer and activator of transcription
MAVS: Mitochondrial antiviral-signaling protein
NTD: N-terminal domain
ORF: Open reading frame
PLpro: Papain-like protease
PAMP: Pathogen-associated molecular pattern
PPs: Polyprotein
RTC: Replicates transcriptase complex
RIG-I: Retinoic acid-inducible gene I
RBR: Retinol-binding protein
TLR: Toll-like receptor
TRIM25: Tripartite motif-containing protein 25
Ub: Ubiquitin
WHO: World Health Organization.
